# Diagnostic endoscopic submucosal dissection for a reddish depressed gastric lesion: morphological and pathological upgrading

**DOI:** 10.1055/a-2589-1522

**Published:** 2025-06-04

**Authors:** Tiantian Ma, Rui Cheng, Jianhuang Su, Fandong Meng

**Affiliations:** 1 Department of Gastroenterology, Beijing Friendship Hospital, Capital Medical University, Beijing, China; 2 State Key Laboratory for Digestive Health, National Clinical Research Center for Digestive Diseases, Beijing, China

A 59-year-old woman presented with a 1-year history of acid reflux. One year earlier, she underwent two esophagogastroduodenoscopies (EGDs) at an outside hospital, where biopsies confirmed chronic atrophic gastritis with colonic-type intestinal metaplasia.

Upon presentation at our hospital, standardized EGD ( Fig. 1 ) revealed C3-type atrophy and the HP breath test was positive. A 0-IIa+IIc lesion, approximately 1.0 cm × 1.5 cm, was identified on the greater curvature of the gastric antrum. The lesion had clear boundaries with a reddish, coarse surface, covered by thin mucus and a fine coating ( Fig. 2 ). Magnifying endoscopy revealed irregular, ridge-like glandular pits at the lesionʼs periphery, with twisted, dense microvessels centrally ( Fig. 3 ).

**Fig. 1:**
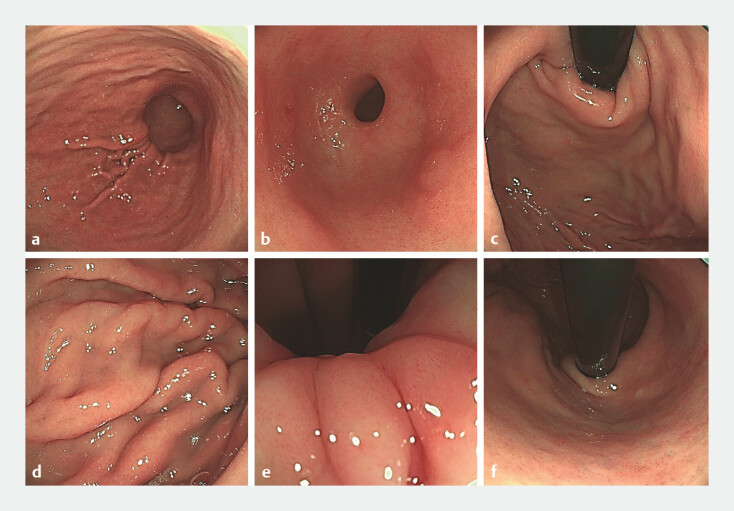
The standardized esophagogastroduodenoscopy revealed the patientʼs background mucosal condition. **a** Gastric body. **b** Gastric antrum. **c** Gastric fundus. **d** Gastric body. **e** Gastric angle. **f** The gastric mucosa exhibits a red-and-white pattern, predominantly white, with the presence of grayish-white nodules.

**Fig. 2:**
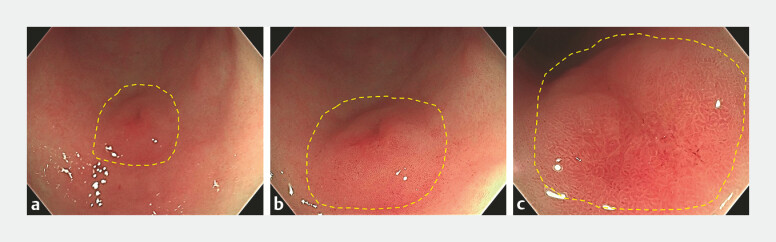
A 0-IIa+IIc lesion, measuring approximately 1.0 cm × 1.5 cm, was observed on the greater curvature of the gastric antrum. **a** The lesion had a generally clear boundary with erythematous mucosa. **b** The surface of the lesion was rough, covered with mucus and a thin coating, without bleeding. **c** The lesion demonstrated good distensibility.

**Fig. 3:**
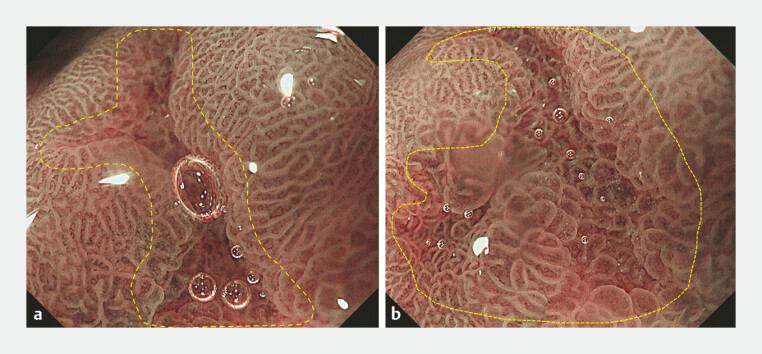
Further characterization of the lesion was performed using magnifying endoscopy. **a** Under magnifying endoscopy, the lesionʼs micro-surface appeared relatively regular. **b** The central depressed area seemed to have twisted, dense, and irregularly sized microvessels.

The patient underwent three EGDs with biopsies within 1 year, all confirming chronic atrophic gastritis with colonic-type intestinal metaplasia. Given the lesionʼs isolated nature and morphological features, alongside the patient’s preference for intervention, we proceeded with diagnostic endoscopic submucosal dissection (ESD).

During ESD, submucosal injection and circumferential mucosal incision were followed by stepwise dissection, resulting in en bloc resection of the lesion ( Video 1 ). The resected specimen measured 31 mm × 28 mm, with a superficial lesion of 8 mm × 5 mm identified on the surface ( Fig. 4 ). Microscopic examination revealed abnormal glandular structures beneath a normal epithelial layer ( Fig. 5 ). The normal epithelial covering made it difficult to determine the true nature of the lesion during endoscopic examination.

The esophagogastroduodenoscopy examination and endoscopic submucosal dissection procedure.Video 1

**Fig. 4:**
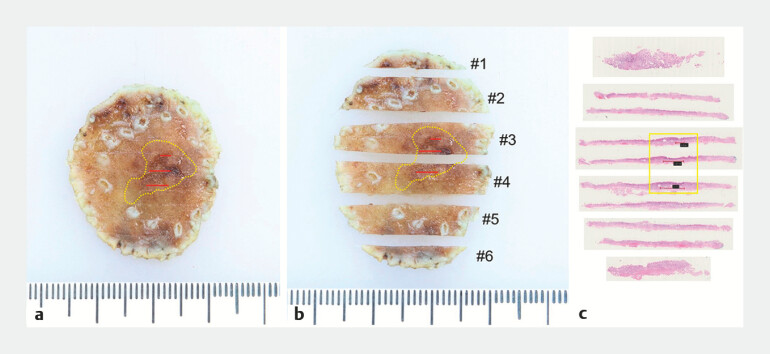
Post-ESD, a piece of gastric antral mucosal tissue was resected, measuring 31 × 28 mm. **a** The surface revealed a 0-IIa + IIc type lesion, measuring 8 × 5 mm. **b** The tissue was divided into six strips, with the lesion concentrated on the third and fourth strips. **c** The tissue strips were examined under a low-power microscope. Abbreviation: ESD, endoscopic submucosal dissection.

**Fig. 5:**
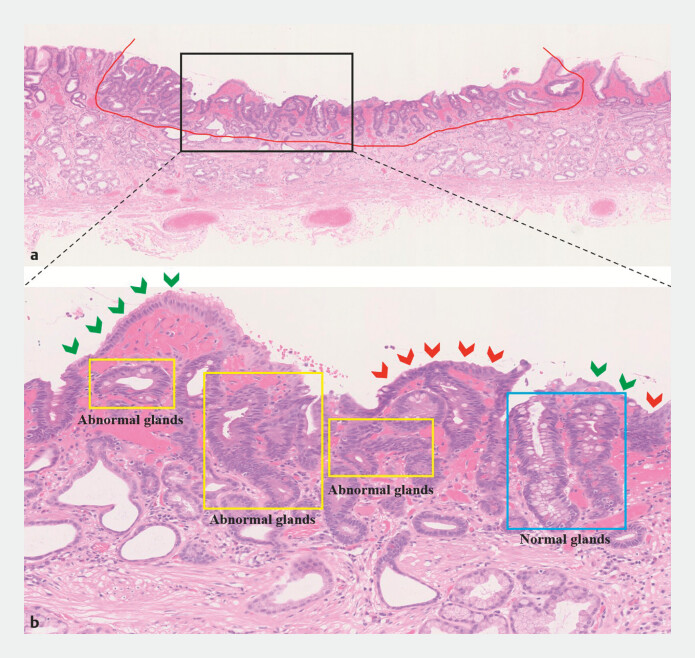
Microscopic examination of the lesion. **a** Microscopic examination revealed a clear demarcation between the lesion and normal tissue. **b** The surface of some of the lesions is covered by normal epithelial structure, while the underlying glands are twisted, fused, and growing horizontally. Green arrow: normal epithelial structure; red arrow: abnormal epithelial structure, with large, deeply stained nuclei visible; blue rectangle: normal glands; yellow rectangle: abnormal glands.

Discrepancies between biopsy and resection pathology are common, with studies reporting a discordance rate of 20.1% 1 . Features such as lesion size >1 cm, reddish, depressed surfaces, and nodular changes are associated with histological upgrading post-resection 1 . This case underscores the importance of diagnostic resection for high-risk lesions, the need for comprehensive assessment in cases of morphological discrepancies, and the critical role of shared decision-making in clinical management.

Endoscopy_UCTN_Code_CCL_1AB_2AD_3AB

## Notes

Endoscopy E-Videos https://eref.thieme.de/e-videos
*E-Videos* is an open access online section of the journal *Endoscopy* , reporting on interesting cases and new techniques in gastroenterological endoscopy. All papers include a high-quality video and are published with a Creative Commons CC-BY license. Endoscopy E-Videos qualify for HINARI discounts and waivers and eligibility is automatically checked during the submission process. We grant 100% waivers to articles whose corresponding authors are based in Group A countries and 50% waivers to those who are based in Group B countries as classified by Research4Life (see: https://www.research4life.org/access/eligibility/ ). This section has its own submission website at https://mc.manuscriptcentral.com/e-videos .
